# Risk Assessment of Shellfish Toxins

**DOI:** 10.3390/toxins5112109

**Published:** 2013-11-11

**Authors:** Rex Munday, John Reeve

**Affiliations:** 1 AgResearch Ltd, Ruakura Research Centre, Private Bag 3123, Hamilton, New Zealand; 2 Ministry of Primary Industries, PO Box 2526, Wellington, New Zealand; E-Mail: John.Reeve@mpi.govt.nz

**Keywords:** shellfish toxins, risk assessment, acute toxicity, chronic toxicity, genotoxicity, absorption and metabolism, toxicity equivalence factors

## Abstract

Complex secondary metabolites, some of which are highly toxic to mammals, are produced by many marine organisms. Some of these organisms are important food sources for marine animals and, when ingested, the toxins that they produce may be absorbed and stored in the tissues of the predators, which then become toxic to animals higher up the food chain. This is a particular problem with shellfish, and many cases of poisoning are reported in shellfish consumers each year. At present, there is no practicable means of preventing uptake of the toxins by shellfish or of removing them after harvesting. Assessment of the risk posed by such toxins is therefore required in order to determine levels that are unlikely to cause adverse effects in humans and to permit the establishment of regulatory limits in shellfish for human consumption. In the present review, the basic principles of risk assessment are described, and the progress made toward robust risk assessment of seafood toxins is discussed. While good progress has been made, it is clear that further toxicological studies are required before this goal is fully achieved.

## 1. Introduction

Humans are exposed to many chemicals in their food. These may be added deliberately, as in the case of food additives, or through contamination by substances found naturally in the environment or by residues from chemicals used in food production, such as pesticides, anthelmintics, antibiotics and growth promotants.

The question arises as to whether such food additives and contaminants have adverse effects on human health. In order to address this question, the process of risk assessment is undertaken.

## 2. Risk Assessment–Definitions and Protocols

Risk assessment comprises four stages: hazard identification, hazard characterisation, exposure assessment and risk characterisation [1,2].

### 2.1. Hazard Identification

Hazard identification involves the identification of the type and nature of adverse effects that the chemical has the inherent capacity to cause in an organism, system or (sub) population [3]. 

### 2.2. Hazard Characterisation

Hazard characterisation involves the quantitative consideration by dose-response evaluation of the nature, relevance and mode of action of adverse effects produced by a chemical. 

Properly-conducted dose-response experiments in animals permit the establishment of No Observable Adverse Effect Levels (NOAELs), the highest tested doses at which no adverse effects are observed. These parameters may be used to identify the Acute Reference Dose (ARfD) and the Tolerable Daily Intake (TDI) of a food contaminant. The ARfD is defined as “the amount of a chemical in food that, in the light of present knowledge, can be consumed in the course of a day or at a single meal with no adverse effects” [3], and relates to the risk of acute effects following a single exposure to the chemical. The ARfD is often derived from acute toxicity studies in animals (and sometimes from toxicological end-points relevant to a single day’s exposure in repeated-dose studies) by application of a safety factor (also known as an uncertainty factor) to the NOAEL. For many years, the default safety factor has been taken as 100, which comprises a 10-fold factor for variation in susceptibility between man and experimental animals and a 10-fold factor to allow for inter-individual variations in response within the human population. The latter factor has been refined to take into account both the kinetics and the target organ sensitivity of individuals, with the conclusion that the 10-fold factor for human variability would cover the vast majority of the population [4]. Different safety factors may be appropriate in some circumstances, however, depending upon such factors as the quality of the toxicological data, the availability of information on effects in humans and the nature of the toxic effects induced [5,6].

Three approved methods for establishing the acute toxicity of a chemical have been described by the Organisation for Economic Cooperation and Development (OECD). These have been published as Guideline 420 (Fixed Dose Procedure) [7], Guideline 423 (Acute Toxic Class Method) [8] and Guideline 425 (Up-and-Down Procedure) [9]. All these procedures have been designed to minimise the number of animals required to estimate the acute toxicity of a chemical, and all require that the protocol described in the OECD Guidance Document on Humane Endpoints [10] is followed. OECD Guideline 425 has the advantage of not only providing an estimate of the acute toxicity of a chemical but also the confidence limits of this estimate, using the computer program associated with this Guideline [11].

Considerable effort has been made toward developing *in vitro* tests to replace *in vivo* acute toxicity evaluations, as undertaken by The Interagency Coordinating Committee on the Validation of Alternative Methods in the United States and the European Centre for the Validation of Alternative Methods. It has been concluded, however, that at the present time, no *in vitro* test method is sufficiently accurate to replace animals for regulatory hazard classification purposes [12,13], although *in vitro* cytotoxicity tests are recommended for determining starting doses for acute toxicity testing in animals, thus reducing the number required for the *in vivo* study [13].

The TDI is defined as “the daily intake of a chemical in food that, in the light of present knowledge, can be consumed every day for a lifetime with no appreciable harmful effects” [3,14], and relates to the situation in which the chemical is present in foodstuffs for prolonged periods, and may therefore produce chronic adverse effects upon the health of consumers. The TDI is derived from NOAELs determined in toxicological studies in animals and *in vitro*, which include short-term toxicity (repeated daily doses for 14–28 days [15]), sub-chronic toxicity (repeated daily doses for 90 days [16]), chronic toxicity and carcinogenicity studies in animals [17,18,19], investigation of genotoxicity *in vitro* and *in vivo* [20], and reproductive and developmental toxicity. The last-named study involves repeated dosing of the test compound to animals before, during and after gestation [21,22], which may be incorporated into a sub-chronic study [23]. Information is also required on the absorption, distribution, metabolism and excretion of the chemical. An understanding of the biochemical mechanism whereby a toxic chemical exerts its adverse effects is also very valuable in risk assessment, since this may explain the target tissue of the chemical and could also indicate if particular groups of individuals are likely to be especially vulnerable to its toxic effects. 

Studies in animals on hazard characterisation of toxins should be conducted using the route of administration appropriate to consumers. For food additives and contaminants, this is obviously the oral route.

### 2.3. Exposure Assessment

Exposure assessment involves the estimation of the amount of the chemical consumed by humans.

### 2.4. Risk Characterisation

Risk characterisation is the combined consideration of hazard identification, hazard characterisation and exposure assessment to estimate the degree of risk that a contaminant poses and the level that is predicted to cause no adverse effects in humans.

## 3. The Problem of Shellfish Toxins

Poisoning of humans by shellfish has been recognised for many years. From early times, Native Americans living on the west coast of North America were aware of the danger of eating shellfish when bioluminescence, indicating the presence of an algal bloom, was observed in the sea [24]. The first recorded report of death from shellfish appears to be that of George Vancouver, captain of the sloop “Discovery”, during exploration in 1793 of the area now known as British Columbia [25]. The description of the symptoms given by the ship’s surgeon was consistent with paralytic shellfish poisoning, now known to be caused by saxitoxin and derivatives. Other syndromes were similarly named on the basis of symptomology—diarrhetic shellfish poisoning, caused by okadaic acid and the dinophysistoxins, neurotoxic shellfish poisoning, caused by the brevetoxins, and amnesic shellfish poisoning, induced by domoic acid. Many more toxic compounds have now been identified as shellfish contaminants, including azaspiracids, ciguatoxins, tetrodotoxin, yessotoxins, pectenotoxins, the cyclic imines (gymnodimines, spirolides, pinnatoxins, pteriatoxins, prorocentrolide, spiro-prorocentrimine and symbioimines) and the palytoxins, which include palytoxin itself and the ovatoxins [26,27]. In 2000, it was estimated that 60,000 individuals suffer intoxication by shellfish toxins each year, with a 1.5% mortality rate [28]. The major symptoms of shellfish poisoning in humans are summarised in Table 1.

**Table 1:** Symptoms of shellfish poisoning in humans.

Toxin class	Reported effects in humans	Reference
Azaspiracids	Nausea, vomiting, diarrhoea, abdominal pain.	[29]
Brevetoxins	Nausea, vomiting, diarrhoea, chill, sweating, dysaesthesia, hypotension, paraesthesia of lips, face and extremities, cramps, paralysis, seizures and coma after ingestion. Rhinorrhoea, cough, bronchoconstriction after inhalation.	[3,30]
Ciguatoxins	Vomiting, diarrhoea, bradycardia, hypotension, pruritis, arthralgia, myalgia, hyporeflexia, dysphagia, ataxia, paralysis.	[31]
Cyclic imines	None.	[32]
Domoic acid and derivatives	Vomiting, diarrhoea, abdominal pain, confusion, memory loss, seizure, coma, death.	[33]
Okadaic acid and derivatives	Nausea, vomiting, diarrhoea, abdominal pain.	[34]
Palytoxin and derivatives	Nausea, vomiting, myalgia, rhabdomyolysis, renal failure and death after ingestion. Rhinorrhoea, cough, bronchoconstriction after inhalation.	[35]
Pectenotoxins	None.	[36]
Saxitoxin and derivatives	Nausea, paraesthesia, tachycardia, muscular paralysis, respiratory failure, death.	[37]
Tetrodotoxin and derivatives	Nausea, vomiting, diarrhoea, abdominal pain, paraesthesia, muscular paralysis, respiratory failure, death.	[27]
Yessotoxin and derivatives	None.	[38]

Shellfish toxins are derived mainly from phytoplankton and bacterioplankton. These organisms are ingested by filter-feeding shellfish, zooplankton and herbivorous fishes, and the toxins may accumulate within the tissues of these animals. These act as vectors for human intoxication either directly, as in the case of shellfish, or indirectly through transfer via the food web to crustaceans and carnivorous fishes [39,40,41]. There is presently no practicable means of preventing uptake of the toxins by shellfish or of removing the toxins from the shellfish after harvesting, so robust risk assessment of these substances is required so that appropriate regulatory limits can be set in order to protect the health of consumers.

A particular problem with the risk assessment of shellfish toxins is the fact that, within a toxin group, shellfish may be contaminated with many analogues of the parent compound. For example, 57 analogues of saxitoxin, produced by dinoflagellates and cyanobacteria, had been described up to the year 2010 [42], and further research may lead to the discovery of many more. The situation is further complicated by the fact that some toxins are metabolised after consumption by predators, yielding other potentially toxic substances [43,44,45,46]. For risk assessment, an evaluation of the contribution of the components of the toxin mixture to the total toxicity is required, with the establishment of toxicity equivalence factors, as discussed below. 

The situation is further complicated by the fact that shellfish may be contaminated by toxins from more than one group, and the effect of one class of compound on the toxicity of another class must be considered. 

The risk assessment of shellfish toxins is thus a formidable task, and, as outlined below, the toxicological data required for hazard characterisation is often inadequate, and in many cases ARfDs have been set on the basis of acute toxicity in animals by intraperitoneal injection, which is clearly not relevant to the human situation. 

## 4. Hazard Identification of Shellfish Toxins

The toxicity to humans of the saxitoxins, brevetoxins, azaspiracids, ciguatoxins, palytoxin and derivatives, okadaic acid and derivatives, tetrodotoxin and domoic acid has been well characterised. In contrast, while the cyclic imines, the yessotoxins and the pectenotoxins have been shown to be acutely toxic to animals, there is presently no evidence that these substances have caused toxic effects in humans [3]. The last-named substances are currently regulated, largely on the basis of their toxicity in intraperitoneal mouse bioassays. However, the Codex Alimentarius Commission has recommended that, on the basis of presently-available information, neither the pectenotoxins nor the yessotoxins should continue to be regulated [47]. 

## 5. Hazard Characterisation of Shellfish Toxins

### 5.1. Acute Reference Doses of Shellfish Toxins

No acute reference doses have been proposed for the brevetoxins [3,30] or the cyclic imines [3,32]. Based on human data, acute reference doses for the azaspiracids [3,29], domoic acid [3,33], okadaic acid and derivatives [3,34], and the saxitoxins [3,37] have been recommended, while proposed ARfDs for palytoxin [35], the pectenotoxins [36] and the yessotoxins [3,38] were based on experimental results in animals. Some of these recommendations are open to question. 

The proposed ARfD for the pectenotoxins was based on results published by Ishige *et al.* [48], who showed intestinal fluid accumulation and villous damage in a single mouse dosed by gavage with pectenotoxin-2 of unspecified purity at a dose of 250 µg/kg. Microalgae of the genus *Dinophysis* produce not only pectenotoxins but also the diarrhetic shellfish toxins, okadaic acid and its derivatives. Algal extracts and extracts of shellfish that had consumed *Dinophysis* therefore inevitably contain both groups of toxins and separation of the pectenotoxins from the diarrhetic toxins is not an easy matter [49]. Since the intestinal effects seen by Ishige *et al.* are characteristic of okadaic acid and its derivatives, it is possible that these effects were due to use of an impure sample. This conclusion is supported by the observation that oral administration to mice of a fully characterised sample of pectenotoxin-2 at a dose of 5000 µg/kg caused no diarrhoea or any other toxic effects [49]. It is likely, therefore, that the suggested ARfD for the pectenotoxins is too low.

The ARfD for yessotoxin was based on histological and ultrastructural changes in the hearts of mice dosed orally with this substance. Slight intracellular oedema was seen by light microscopy in mice dosed by gavage with yessotoxin at 10 and 7.5 mg/kg. Similar changes were seen in a control mouse. By electron microscopy, swelling of myocardial cells and separation of muscle fibres and mitochondria were seen at doses as low as 2.5 mg/kg [50]. Similar ultrastructural changes were observed after an oral dose of 1 or 2 mg/kg of yessotoxin [51], although in a later study, no ultrastructural changes were seen in the hearts of mice given a single oral dose of yessotoxin at 1 or 5 mg/kg [52]. Ultrastructural changes have also been reported in the hearts of mice dosed for 7 days with yessotoxin at 2 mg/kg/day, although no cardiac changes were observed by light microscopy [53]. In contrast, no ultrastructural alterations were seen in the hearts of mice dosed orally with yessotoxin at up to 5 mg/kg, 7 times over a 3-week period [54]. The inconsistent reports of histologically-observable cardiac changes, and the fact that similar changes were seen in a control mouse, call into question the significance of these results, and, as discussed previously [55], the toxicological significance of ultrastructural changes can sometimes be unclear, particularly if effects are not observed by other approaches at higher doses. No deaths were recorded at oral doses of yessotoxin up to 54 mg/kg [56], and examination of the hearts of animals dosed at such levels by both light and electron microscopy should resolve this point. As it stands, the ARfD of yessotoxin may again be set too low.

In their evaluation of palytoxin, the EFSA CONTAM Panel [35] paid particular attention to an experiment that purported to show absorption of palytoxin across the buccal mucosa of mice, with consequent harmful effects in tissues, particularly in the lung [57]. Because of this, they introduced an additional safety factor of 10 because of a perceived increase in the likelihood of palytoxin absorption in humans. In the reported experiment, a solution of palytoxin was administered sublingually to mice at a dose of between 172 and 223 µg/kg. This method of administration is not relevant to the human situation, however, since palytoxin will not be ingested as a solution but as a mixture with food, and when palytoxin was given to mice as a mixture with food, no effects were seen at a dose of 2500 µg/kg [58], suggesting that palytoxin does not undergo significant buccal absorption when administered via a relevant route. Recent studies [59] showing no significant passage of palytoxin across a Caco-2 monolayer *in vitro* support this view. It has been suggested that the observed effects of sublingual palytoxin were due to inhalation of a small amount of the administered solution [60]. It would appear that the extra 10-fold safety factor imposed by the EFSA CONTAM Panel is unnecessary.

A major problem in the setting of ARfDs for shellfish toxins using animal data is that relatively little information is available on their oral toxicity. Often, the oral toxicity of the major toxin is known, but only intraperitoneal data are available for congeners present at lower concentration. In the case of azaspiracids [29], okadaic acid and derivatives [34], the pectenotoxins [36] and the yessotoxins [38] it has been assumed that the relative oral toxicity of derivatives is accurately predicted by their relative toxicity by intraperitoneal injection. Furthermore, it has been assumed [3,37] that the relative acute toxicity of saxitoxin and derivatives is predicted by their behaviour in the mouse bioassay. As discussed in Section 7, these assumptions are not valid.

### 5.2. Tolerable Daily Intake of Shellfish Toxins

Blooms of toxigenic algae are episodic, and at their height may result in high concentrations of toxins in shellfish, sufficient to cause illness or death after a single meal. In this situation, the ARfD, based on acute toxicity studies, is appropriate for risk assessment. After the dispersal of an algal bloom, toxin will be lost through depuration, ultimately reaching levels insufficient to cause acute effects. It should be noted, however, that many toxins are but slowly depurated, and, while levels may be below the regulatory limit based on the ARfD, residues may still remain. For example, razor clams have been shown to retain domoic acid for more than a year [61]. Similarly, measurable amounts of saxitoxin and derivatives [62,63], okadaic acid [64,65], pectenotoxin [65], gymnodimine [66,67], azaspiracids [68,69], brevetoxins [70,71] and yessotoxins [72] have been shown to remain in shellfish for months after cessation of exposure to toxigenic algae. Since many individuals consume seafood on a regular basis, the long-term presence of toxins, albeit at below the regulatory limits relating to acute effects, raises the question as to the possible need to set TDIs for such toxins.

At present, no repeat-dosing studies in animals using approved protocols have been reported, and while some information on the effects of repeated doses of certain toxins is available, the EFSA CONTAM Panel has concluded that insufficient evidence is available to set a TDI for any shellfish toxin. 

No behavioural or histological effects were seen in rats dosed by gavage with domoic acid at 0.1 or 5.0 mg/kg/day for 64 days [73]. However, examination of the brains of a subset of these rats by electron microscopy revealed neuronal damage in the hippocampus of animals receiving the higher dose of the test substance [74]. No histological changes were seen in the tissues of Cynomolgus monkeys dosed for 15 days with domoic acid at 0.5 mg/kg/day and then 0.75 mg/kg/day for a further 15 days [75], but no ultrastructural examination of the brains of these animals was undertaken. 

Okadaic acid dosed to mice at 1 mg/kg/day for up to 7 days caused death in 2 out of 5 animals. Surviving mice killed after 7 days showed biochemical changes indicative of liver damage, and ulceration and submucosal inflammation of the forestomach were observed [53]. There is evidence that the toxicity of certain ciguatoxins may be cumulative. Intraperitoneal injection of Pacific CTX-1 at 0.26 mg/kg caused no deaths, but after a second dose at the same level 3 days after the first, 30% of a group of mice died [76]. No pathological changes were seen in mice after a single intraperitoneal dose of Pacific ciguatoxin-1 or Pacific ciguatoxin-4C at 0.1 mg/kg, but single-cell necrosis in the heart was observed by light microscopy after this dose was given daily for 15 days [77].

Mice dosed intraperitoneally with a shellfish extract containing saxitoxin and derivatives were reported to be more susceptible than control animals to a second dose given 12–24 hours later [78]. In contrast, it has been reported that oral administration of a sublethal dose of saxitoxin decreased the toxicity of a subsequent dose given 14 days later [79].

### 5.3. Genotoxicity of Shellfish Toxins

Brevetoxin-2 and brevetoxin-6 [80], palytoxin [81], tetrodotoxin [82] and okadaic acid [83] have been tested for mutagenicity in the standard Ames test, using tester strains TA 98 and TA 100, with or without metabolic activation. All were negative. No conventional mutagenicity assays on domoic acid, azaspiracids, cyclic imines, saxitoxins, yessotoxins or pectenotoxins have been reported.

DNA strand breaks and chromosomal aberrations were observed in cells incubated *in vitro* with brevetoxins [84,85]. DNA strand breaks have also been observed with domoic acid [86], although this compound was not genotoxic in V79 Chinese hamster lung fibroblasts, with or without metabolic activation [87]. Okadaic acid was mutagenic in Chinese hamster lung cells [83] and DNA damage has been reported in some cell lines incubated with this substance, but not in others [88,89,90,91,92]. Micronucleus formation has also been recorded in several cell lines incubated with okadaic acid *in vitro* [93,94,95]. It has been suggested [34] that some of these effects may reflect the cytotoxic activity of okadaic acid, rather than a specific action on DNA.

Brevetoxin-2 caused DNA breaks in the livers of rats dosed intratracheally with brevetoxin-2 [80]. Tetrodotoxin was negative in the mouse bone-marrow micronucleus test [82], but no other shellfish toxins appear to have been tested in the recommended standard tests for *in vivo* genotoxicity.

### 5.4. Carcinogenicity of Shellfish Toxins

No chronic toxicity or carcinogenicity studies employing standard tests have been reported for any of the shellfish toxins.

Ito *et al.* [96] dosed AZA-1 to mice by gavage at 1, 5, 20 or 50 μg/kg twice a week, over a period of up to 20 weeks. At the highest dose, all mice died or were killed *in extremis* within 40 doses, and at the 20 µg/kg dose, 30% of the mice died. Surviving mice were subsequently left untreated for up to 3 months, after which time they were killed. Four lung tumors were observed in these mice at necropsy. In order to further investigate the possible pulmonary carcinogenicity of AZA-1, groups of mice were dosed by gavage with this substance at dose-levels of between 5 and 20 µg/kg, once or twice per week, for a total of between 20 and 40 doses. The majority of these mice were killed after 8 months. No tumors were seen at this time. The remaining 20 mice were kept for a further 4 months, when 9 tumors (seven lung tumors and two malignant lymphomas) were found [97]. While these observations are widely cited as indicating a carcinogenic effect of AZA-1, the carcinogenicity of this substance cannot be considered proven. The CD-1 mice used in these experiments have a high spontaneous incidence of lung tumors, as do many other strains of laboratory mouse [98,99]. In their review of the use of the mouse in carcinogenicity testing, Grasso and Crampton [100] concluded that “the induction of pulmonary tumors in the mouse has limited relevance in terms of human carcinogenic hazard”. The EFSA CONTAM Panel [29] also concluded that the observations of Ito *et al.* with regard to the pulmonary carcinogenesis of azaspiracids are of limited relevance.

### 5.5. Tumor Promotion by Shellfish Toxins

Repeated application of okadaic acid, dinophysistoxin-1 or palytoxin to mouse skin promoted tumour formation following initiation with 7,12-dimethylbenz[*a*]anthracene [101,102]. Okadaic acid, when administered via the drinking water, also acted as a tumour promoter in the rat glandular stomach after initiation with *N*-methyl-*N’*-nitro-*N*-nitrosoguanidine [103]. Neither the okadaic acid derivatives nor palytoxin were initiators of cancer.

In view of the tumour-promoting activity of these substances, it could be argued that an additional safety factor should be employed in order to take account of this effect. However, many substances to which humans are regularly exposed, such as detergents, fatty acid esters, citrus oils, saccharin and medium-chain hydrocarbons, have been shown to be tumour promoters in animals [104,105,106,107,108], suggesting that the shellfish contaminants are unlikely to significantly increase human exposure to promoters. Furthermore, there is no epidemiological evidence that exposure to tumour promoters leads to an increase in cancer incidence [109], and tumour promotion is considered to be a threshold effect. Since a common property of tumour promoters is their ability to induce inflammation via irritation [110,111,112], the promoting activity of okadaic acid, dinophysistoxin-1 and palytoxin may simply be due to their irritant effect [102,113,114], which is unlikely to be expressed following exposure to small amounts of these substances in food. An extra safety factor for the okadaic acid derivatives and palytoxin with regard to tumour promotion therefore appears to be unnecessary.

### 5.6. Reproductive and Developmental Effects of Shellfish Toxins

No evaluation of the potential reproductive and developmental toxicity of shellfish toxins by use of standard tests has been reported. Developmental effects have, however, been described in animals dosed with domoic acid. Newborn pups from pregnant mice injected intravenously with domoic acid at gestational day 13 showed no brain lesions, but by post-natal day 14, severe neuronal damage was observed in the hippocampus, which continued to increase in severity until the end of the observation period, at post-natal day 30 [115]. When tested at 11 weeks of age, male, but not female, mice from dams given intraperitoneal injections of domoic acid at various stages of gestation showed severe impairment of learning and memory [116]. In rats, prenatal exposure to domoic acid led to persistent behavioural changes at up to 13 weeks in both males and females [117,118].

Okadaic acid was detected in the foetuses of pregnant mice dosed orally with this substance at day 11 of gestation [119], and transfer of brevetoxin-3 to the placenta and foetuses of pregnant mice was demonstrated after intra-tracheal administration at day 15 or 16 of gestation [120], although possible effects on the offspring were not examined in these studies.

It appears that ciguatoxins can cross the human placenta. A woman who ate a ciguateric fish 2 days before the expected birth date of her child noticed a pronounced increase in foetal movement. The baby was born with left-sided facial palsy and possible myotonia of the muscles of the hands, although the child appeared normal at 6 weeks of age [121]. Increased foetal movements were also observed after consumption of a ciguateric fish by a woman who was 16 weeks pregnant. At term, she gave birth to a healthy baby, which developed normally over the subsequent 10-month observation period [122].

### 5.7. Inhalation Toxicity of Shellfish Toxins

Harmful effects in humans through ingestion of brevetoxin-contaminated seafood are relatively uncommon, but more widespread exposure of humans to brevetoxins occurs through inhalation. The *Karenia* species that produce brevetoxins are fragile, and readily break under the action of waves. With an inshore wind, beachgoers and persons living close to the beach may be exposed to aerosols containing brevetoxins, in which BTX-2 and BTX-3 predominate [123]. Such exposure leads to irritation of the conjunctiva and of the respiratory tract [124]. Animal studies showed a slight increase in the number of pulmonary alveolar macrophages in rats exposed to aerosolised BTX-3 through nose-only inhalation for 5 or 22 days, but there was no evidence of cytotoxicity or inflammation in the lungs. No histological lesions were observed in the nose, liver or bone marrow, and in these studies, no neuronal damage or loss was detected in sections of the hippocampus or cerebellar cortex [125,126]. Later work, however, showed neuronal damage in a specific area of the cerebrum, the retrosplenial cortex, in mice exposed to BTX-3 by inhalation on two consecutive days [127].

### 5.8. Metabolism and Disposition of Shellfish Toxins

Azaspiracid-1 [128], brevetoxin-3 [129], Pacific ciguatoxin-1 [130], okadaic acid [131], and saxitoxin and derivatives [132,133] are well absorbed from the gastrointestinal tract of animals after oral administration and are subsequently distributed among internal organs. The rapid onset of signs of toxicity observed after oral administration of gymnodimine [134], spirolides [135] and pinnatoxins [136] to mice suggests that these cyclic imines are also rapidly taken up from the intestine. In contrast, it is estimated that only 1.8% of an oral dose of domoic acid is absorbed from the gastrointestinal tract of the rat [73]. A mixture of pectenotoxin-2 and pectenotoxin-2-seco acid was largely excreted unchanged in the faeces of mice after oral administration, and only traces of these substances were found in the livers of these animals after 6 hours, and none was detectable in tissues after 24 hours [137]. Yessotoxin also appears to be poorly absorbed by animals. One day after an oral dose of this compound, the total amount in the internal organs of mice accounted for less than 0.1% of that administered, although high levels were found in the ileum and colon [52].

Less than 2% of an oral dose of azaspiracid-1 remained in the internal organs of mice 24 hours after dosing [128]. 13-Desmethyl spirolide C and 13,19-didesmethyl C were rapidly removed from the blood after oral administration [138], and the plasma half-life of domoic acid was estimated as 21 minutes in rats [139]. The rapid recovery of animals dosed with sub-lethal amounts of gymnodimine [134], spirolides [135] and pinnatoxins [136] are consistent with rapid excretion and/or detoxification of these substances. In contrast, brevetoxin-3 was shown to remain in rat tissues for at least 8 days after oral administration [129], Pacific ciguatoxin-1 was detectable in the liver, muscle and brain of rats 4 days after oral administration [130], and okadaic acid was detectable in the heart, lungs, liver and kidneys for up to 2 weeks after an oral dose, and in the small and large intestine for 4 weeks [138]. Similarly, while 58% of an intravenous dose of saxitoxin was excreted in the urine within 24 hours after administration, elimination of the remainder was slow, and this substance was still detectable in the urine 6 days after administration [141]. Likewise, 28% of an intravenous dose of the reduced derivative of saxitoxin, saxitoxinol, remained in tissues 6 days after administration [142]. It has been suggested that okadaic acid [131] and saxitoxinol [142] undergo enterohepatic circulation.

The major urinary metabolites of brevetoxin-2 after intraperitoneal injection in rats are the cysteine and cysteine sulphoxide conjugates [143], while brevetoxin-2, brevetoxin-3 and oxidized and reduced brevetoxins were found in the urine of humans consuming contaminated shellfish [144,145]. In humans, there is evidence that dinophysistoxin-3 is saponified to dinophysistoxin-1 [146], and that *N*-oxidation of saxitoxin and derivatives occurs, together with decarbamoylation and desulphation [147,148,149].

### 5.9. Mechanism of Toxicity of Shellfish Toxins

There is good evidence that the cerebral toxicity of domoic acid reflects an excitotoxic response, due to activation of kainic acid and α-amino-3-hydroxy-5-methyl-4-isoxazolepropionic acid receptors in neurones [150].

Brevetoxins [151] and ciguatoxins [31,152] activate site 5 of the α-subunit of voltage-gated sodium channels, while saxitoxin [153] and tetrodotoxin [154] interact with site 1 of these channels. This results in blockade of ion conduction and generation of action potentials, resulting ultimately in loss of neuromuscular function and muscular paralysis [153]. Gymnodimine [155,156], spirolides [156,157] and pinnatoxins [158] block nicotinic acetylcholine receptors, which again leads to muscular paralysis. Furthermore, brevetoxins [159,160], ciguatoxin-1 [161], gymnodimine [155] and pinnatoxins [162] have been shown to block neuromuscular transmission in the rat phrenic nerve-hemidiaphragm preparation. The effects on neuromuscular transmission are consistent with the respiratory paralysis induced by these compounds in animals, which leads to death by asphyxia.

Palytoxin binds to membranal Na^+^/K^+^-ATPase, converting the ion pump into a non-specific ion channel, thus permitting the uncontrolled transport of ions across the plasma membrane. There is evidence that such disruption of ionic equilibria is responsible for the toxic effects of palytoxin *in vitro* [163], and it is widely stated in the literature that this is also responsible for the *in vivo* effects of this substance. There is, however, no evidence that interaction with Na^+^/K^+^-ATPase is involved in the toxic effects of palytoxin in animals [60]. Interestingly, like brevetoxins, ciguatoxin-1, cyclic imines and saxitoxin, palytoxin causes death by respiratory arrest, and this compound has also been shown have the same effect in the rat phrenic nerve-diaphragm preparation as these compounds [164], suggesting that palytoxin could similarly exert its toxic effects through inhibition of neuromuscular transmission.

Okadaic acid has been shown to inhibit protein phosphatases *in vitro*. In 1990, it was suggested [165] that this effect could be responsible for the diarrhoeagenicity of okadaic acid. It is now widely stated in the literature, without any supporting evidence, that all the toxic effects of okadaic acid are due to protein phosphatase inhibition. No such inhibition has been demonstrated *in vivo*, however, nor has the pathway from enzyme inhibition to toxicity been elucidated, and the relative inhibitory activities of okadaic acid derivatives *in vitro* do not correlate with their toxicity *in vivo* [166]. 

The mechanism(s) of toxicity of the azaspiracids, the pectenotoxins and the yessotoxins are unknown.

### 5.10. Exposure Assessment of Shellfish Toxins

Exposure of consumers to toxins is a function of the amount of shellfish eaten by consumers and the amount of the toxin in the shellfish.

#### 5.10.1. Amount of Shellfish Eaten by Consumers

In the past, a “standard portion” of shellfish of 100 g was often used in risk assessment. The FAO/IOC/WHO Expert Consultation [3] considered that this figure was inadequate, and indicated that a portion of 250 g would cover 97.5% of consumers in most countries for which data were available. A re-evaluation of shellfish intake in Europe was undertaken by the EFSA CONTAM Panel in 2010 [167], who concluded that a portion size of 400 g of shellfish should be used in risk assessment, in order to allow for high consumers.

#### 5.10.2. Assessment of the Amount of Toxin in Shellfish

The mouse bioassay (MBA) has been widely used for determination of toxins in shellfish. This assay involves injection of shellfish extracts to mice by the intraperitoneal route. The end-point of the MBA is death. The MBA has been strongly criticised on the grounds of inhumanity [168,169,170] and on the basis of non-specificity, false positives, interference by certain metals, effects of sex, strain and weight of the animals, the effects of pH of the injected solution and poor inter-laboratory agreement [169,171,172,173]. Until 2011, the MBA was the reference method for assay of lipophilic toxins (okadaic acid and derivatives, pectenotoxins, yessotoxins and azaspiracids) [174], but work on validation of alternative assays led to adoption of LC-MS/MS by the European Union as the reference method for such toxins. This decision took effect on 1 July 2011, and the mouse bioassay will be phased out by 31 December 2014 [175].

The LC-MS/MS assay offers great advantages in terms of specificity and versatility, although some problems remain for its application. This technique is capable of identifying a specified toxin in a shellfish extract, but for quantitation, the response of the instrument to this toxin must be established, and this requires calibration with a certified standard. Few certified standards are available at present, and further work on the provision of such standards is urgently needed. A second problem is that even if the quantity of each component of a mixture of toxins in a shellfish extract is accurately determined, the relative toxicity of each substance must be known for the toxic potential of the extract to be assessed. This requires the determination of appropriate toxicity equivalence factors, as discussed below.

## 6. Toxicity Equivalence Factors for Shellfish Toxins

With the advent of chemical methods of analysis, the amounts of many toxin congeners in a shellfish sample will be available. For risk assessment, the contribution of each constituent to the overall toxicity of the sample needs to be quantitated by the use of toxicity equivalence factors (TEFs), which indicate the toxicity of each compound in relation to a reference compound. Ideally, the TEFs of all congeners present in shellfish should be individually assessed. In this way, assuming that toxicity is additive, the total toxic potential of the mixture can be calculated. With the large number of congeners identified for many classes of toxin, however, this is generally not a practicable proposition, and it has been suggested [3] that congeners of toxins present at 5% or less of the total amount of toxin in the shellfish should be considered of no toxicological significance.

For TEFs in relation to acute toxicity, data on median lethal doses are employed. The TEFs presently used in Europe for derivatives of okadaic acid, azaspiracid, yessotoxin and pectenotoxin are based on acute toxicity by intraperitoneal injection [171]. This route of administration is clearly irrelevant to the human situation. Furthermore, because of different absorption and systemic distribution rates throughout the body, and possible first-pass metabolism in the liver, oral toxicity cannot be predicted on the basis of intraperitoneal toxicity unless good data on toxicokinetics are taken into account. The impact of toxicokinetics is illustrated by the data of Table 2, which shows that the ratio between an intraperitoneal LD_50_ and that determined by oral administration, whether by gavage or by feeding, varies over a wide range, even among congeners within a toxin group.

**Table 2:** Comparison of median lethal doses of certain shellfish toxins by oral administration and by intraperitoneal injection.

Compound	Ratio of toxicity by gavage to toxicity by i.p. injection	Ratio of toxicity by feeding to toxicity by i.p. injection	Reference
Saxitoxin	43	115	[176]
Neosaxitoxin	79	142	[176]
GTX-1&4	110	233	[176]
Yessotoxin	>180	-	[56]
Palytoxin	708	>3500	[58]
Gymnodimine	8	>78	[134]
Spirolide A	15	35	[135]
13-Desmethyl spirolide C	23	145	[135]
Pinnatoxin E	49	-	[136]
Pinnatoxin F	2	4	[136]

The situation is even worse with saxitoxin and derivatives, for which TEFs have been derived from data obtained in the MBA. It must be remembered that the MBA is an assay, not a toxicological parameter. The saxitoxin MBA was developed in the 1930’s by Sommer and Meyer [76], who determined the relationship between the dose of pure saxitoxin administered to mice by intraperitoneal injection and the time to death of the animals. The dose-death time curve is exponential in form, but the slope at death times between 5 and 7 minutes is close to linear, and it is this part of the curve that is employed for the MBA [177]. For assay of the quantity of saxitoxin in a solution, individual mice are injected with various dilutions of the solution until a death time of 5–7 minutes is achieved. The amount of saxitoxin, expressed in “Mouse Units” (MUs) can then be read off from the dose-death time table established by Sommer and Mayer [177], and the specific activity of saxitoxin, defined as the number of MUs contained in 1 µmole of the toxin, can be calculated. The saxitoxin MBA has been applied to many saxitoxin derivatives, and the supposed toxicities of these have been derived by comparing their specific activities with that of saxitoxin itself [37]. In this process, it is assumed that the dose-death time curves for all the saxitoxin derivatives are the same as that for saxitoxin itself, although there is no evidence in support of this assumption. Furthermore, it is not known how the results of the bioassay relate to the median lethal doses of the saxitoxin derivatives.

This problem has recently been explored for saxitoxin and four of its derivatives, all of which were certified standards. The MBA was conducted according to the AOAC official method and the LD_50_ determined according to OECD Guideline 425. As shown in Table 3, there is no correlation between the specific activity of the saxitoxin derivatives in the MBA and their median lethal doses, reflecting the fact that the dose-death time curves for these substances, particularly neosaxitoxin and gonyautoxins 1&4, are quite different to that of saxitoxin itself [176]. 

**Table 3:** Comparison of specific activities of the saxitoxin derivatives, determined by the MBA, and their acute toxicity by i.p. injection.

Compound	Relative specific activity by the MBA	Relative LD_50_ by i.p. injection
Saxitoxin	1.00	1.00
Neosaxitoxin	1.16	3.12
Decarbamoylsaxitoxin	0.64	0.79
Gonyautoxins 1&4	1.02	1.90
Gonyautoxins 2&3	0.60	0.76

## 7. Discussion

The problem of shellfish toxicity appears to be escalating throughout the world. Increases in the frequency and severity of blooms of harmful algae have been reported, and non-indigenous toxigenic organisms are now being found in many areas. Such changes may reflect climate change (possibly due to increases in atmospheric greenhouse gases), eutrophication of coastal waters, loss of coastal biodiversity and transport of species from endemic to non-endemic areas through discharge of ballast water or movement of shellfish stocks from one area to another [28,178,179,180,181,182]. Furthermore, new toxigenic algal species, new toxin groups and new congeners of known toxins are continually being discovered. Appropriate risk assessment of shellfish toxins therefore becomes increasingly important.

Robust data on acute oral toxicities are required for the establishment of relevant TEFs [183], which are becoming more important with the increasing acceptance of chemical methods for analysis of toxins in shellfish. As discussed previously [171,184], the comparative oral toxicities of these compounds should be determined using approved methods and with certified standards. While it is assumed that the effects of the various congeners within a toxin group are additive, there is presently no information to support such an assumption. Dose additivity should be tested following exposure to combinations of toxin analogues [171].

Acute oral toxicity has generally been determined after administration by gavage, which, due to the structure of the rodent stomach and the consistency of its contents, can give an artefactually high estimate of acute toxicity [58,134]. The Codex Alimentarius Commission [47] recommended that administration by feeding, rather than by gavage, should be employed in acute oral toxicity tests with shellfish toxins, since this route is most relevant to the human situation. This involves ingestion by the test animals of food spiked with the test compound, which, for acute toxicity studies, must be completely consumed within seconds. Mice fasted overnight will rapidly eat a small piece of mouse food, either dry or moistened with water, to which a known amount of the toxin may be added. Alternatively, after a period of training, mice will readily eat cream cheese or a mixture of low-fat peanut butter (53%), casein (10%) and sucrose (37%), and this technique has the advantage that the food is rapidly and enthusiastically eaten by both fasted and fed mice, so that fasting is not necessary for acute toxicity determinations. In studies on the acute toxicity of pinnatoxin F, no differences in response were observed among fasted animals given this substance on mouse food or in the cheese or peanut butter mix [136]. 

Studies on the absorption and distribution of toxins after administration of a single oral dose are very valuable. It is likely that the relatively low oral toxicity of yessotoxins, pectenotoxins and palytoxin reflect poor absorption from the gastrointestinal tract.

It should be noted that while the oral route is essential for risk assessment of most shellfish contaminants, some algal-derived toxins such as the brevetoxins [124] and the ovatoxins [185,186,187] have been shown to cause adverse effects in humans via inhalation. In this situation, not only oral toxicity but also toxicity by inhalation is required for risk assessment, since the absorption and disposition of toxic substances through the lung are quite different to those via the intestine. The conflicting reports of neurological damage after brevetoxin-3 inhalation require further investigation, and inhalation toxicity studies with the ovatoxins are required.

In view of the prolonged exposure to low levels of shellfish toxins by regular consumers of seafood, consideration should be given by regulatory authorities to the setting of TDIs for these substances. Repeated-dose feeding studies are required for this, and it would be appropriate to begin with 28-day studies, as described in OECD Guideline 407 [15]. Chronic effects could be of particular importance with brevetoxins, ciguatoxins, okadaic acid and saxitoxins, since the slow elimination of these substances by mammals may lead to their build-up in tissues, with the possibility of severe toxic effects. For long-term studies, administration via the diet or drinking water is recommended [18].

Genotoxicity testing by standard methods are required for domoic acid, azaspiracids, saxitoxins, yessotoxins and pectenotoxins.

As discussed above, domoic acid has been shown to cross the placental barrier in animals and cause impairment of learning and memory in the offspring of dams injected with this substance during pregnancy. Studies on foetal and neonatal development are required with other toxins that have been shown to cross the placenta, which include the brevetoxins, okadaic acid and the ciguatoxins.

Risk assessment is usually conducted in young, healthy animals, but it must be recognised that certain disease states and changes associated with ageing in humans may increase susceptibility to certain toxins. It is interesting to note that deaths occurred in the Canadian outbreak of amnesic shellfish poisoning only in individuals over the age of 65 and in individuals with renal disease, hypertension or diabetes [188,189]. For the expression of the neurological damage induced by domoic acid, the toxin must cross the blood-brain barrier. The permeability of this barrier increases during the normal ageing process in humans [190], and the patency of the barrier is compromised by hypertension and diabetes [191,192,193]. Domoic acid is excreted via the kidneys, and prolonged retention of domoic acid in the plasma could have contributed to the death of the individuals with pre-existing renal disease. Renal function also declines with age [194]. For induction of visceral effects, the toxin must be absorbed from the gastrointestinal tract. Absorption of toxins is generally restricted by the mucosal barrier, comprising the mucus layer and the tight junction between mucosal cells. The gastrointestinal epithelium becomes thinner and more fragile with increasing age, and there is a high incidence of ulcers in the elderly [195], which could increase toxicity. Furthermore, alcohol and non-steroidal anti-inflammatory drugs are known to cause epithelial damage in the stomach and intestine of humans [196,197], and this again could increase toxicity. Studies on the effects of age and of damage to the gastrointestinal tract of animals on the toxicity of shellfish toxins would be of interest. 

Shellfish do not always contain only a single class of toxins, and the possibility of an interaction between toxins of different groups must be considered. Some toxins, such as okadaic acid [51], ciguatoxin [198] and azaspiracid [199] themselves damage the epithelium of the gastrointestinal tract, and it is possible that these compounds could increase the absorption of other toxins simultaneously present in the shellfish. Surprisingly, however, Aune *et al.* [200] showed that oral administration of combinations of okadaic acid and azaspiracid-1 led to a decreased extent of absorption of both toxins. Furthermore, Sosa *et al.* [201] showed that simultaneous administration of okadaic acid and yessotoxin to mice did tot lead to any additive toxic effects.

It has sometimes been assumed that a biochemical or physiological change induced by a toxin *in vitro* is responsible for *in vivo* toxicity. It has been argued that the *in vitro* changes suggested to be responsible for the *in vivo* toxicity of okadaic acid [166] and palytoxin [60] are not associated with the toxicity of these compounds. Further work is required on the mechanism of toxicity of palytoxin and okadaic acid, and on those toxins for which the mechanism of toxicity is presently unknown.

More attention needs to be given to the metabolism of toxins in animals and in humans. Dinophysistoxin-1 is more toxic to mice than dinophysistoxin-3 both by injection [202,203] and by oral administration [204,205], and neosaxitoxin is similarly more toxic to mice than saxitoxin [175]. The observations that dinophysistoxin-3 is hydrolysed to dinophysistoxin-1 [146] and that saxitoxin is oxidized to neosaxitoxin [147] in humans are of concern, since these processes would lead to higher toxicity than that predicted from analytical data on the toxin levels in shellfish.

Case studies on individuals known to be poisoned by shellfish contaminants would be very useful in establishing doses that are harmful to humans, and every opportunity should be taken to analyse samples of toxic material eaten by humans.

Much progress has been made in the risk assessment of shellfish toxins, and it would appear that the present ARfDs that have been set to minimise the risk of acute intoxication in humans are effective, although it has been argued that these are inadequate for high consumers [171]. 

Risk assessment is an expensive undertaking, and in order to best utilise scarce resources research should be focused on new compounds or compounds that are known to have adverse effects on human health, such as saxitoxins, azaspiracids, brevetoxins, domoic acid and okadaic acid and derivatives. Compounds such as the yessotoxins and pectenotoxins, which have never been associated with adverse effects on human health, should be of lower priority.
